# The impact of Tai Chi on emotional regulation efficacy and subjective wellbeing in the elderly and the mediating mechanism

**DOI:** 10.3389/fpsyg.2025.1550174

**Published:** 2025-05-23

**Authors:** Shiguang Wang, Yongchao Huang, Xuyan Si, Huakai Zhang, Meiling Zhai, Hongxia Fan, Lin Ding

**Affiliations:** Medical College, Zhengzhou University of Industrial Technology, Zhengzhou, Henan, China

**Keywords:** Tai Chi, geriatric, emotional regulation efficacy, subjective wellbeing, mental health, mediation analysis

## Abstract

**Introduction:**

Against the backdrop of global aging and the enduring impact of the COVID-19 pandemic, mental health issues among older adults have become increasingly prominent. This study aimed to investigate the effects of standardized 24-form simplified Tai Chi training on emotional regulation efficacy and subjective wellbeing in older adults and to verify the mediating role of emotional regulation efficacy between Tai Chi practice and subjective wellbeing.

**Methods:**

The study was conducted in Zhengzhou, China, in autumn 2023. Sixty healthy older adults were randomly assigned to either an experimental group receiving Tai Chi training or a control group maintaining their regular lifestyle. At baseline, both groups were assessed using the Emotional Regulation Efficacy Scale and the Subjective Wellbeing Scale. The experimental group underwent an 8-week Tai Chi program, consisting of five 30-minute sessions per week under professional guidance, while the control group remained unchanged. Post-intervention, all variables were reassessed.

**Results:**

The results indicated significant improvements in emotional regulation efficacy (23.60 ± 2.33 to 26.60 ± 3.10, *p* < 0.001, Cohen’s d = 1.29), positive affect (27.53 ± 2.46 to 29.43 ± 3.12, *p* < 0.001, Cohen’s *d* = 0.77), negative affect (30.70 ± 2.90 to 32.13 ± 3.05, *p* = 0.002, Cohen’s *d* = 0.49), and subjective wellbeing (58.23 ± 5.29 to 61.57 ± 5.04, *p* = 0.000, Cohen’s d = 0.63) in the experimental group compared to baseline, with no significant changes observed in the control group. Between-group comparisons further confirmed the experimental group’s superior improvements across all measured variables. Mediation analysis demonstrated that Tai Chi’s benefits on wellbeing operated through dual pathways: while 83.8% of its total effect on subjective wellbeing was direct, 16.20% [β = 0.637, 95% CI (0.002, 1.731)] was mediated via enhanced emotional regulation efficacy. Notably, 22.27% of Tai Chi’s reduction in negative affect [β = 0.334, 95% CI (0.005, 0.761)] was attributable to this mediating mechanism, whereas no mediation was observed for positive affect—highlighting that Tai Chi’s promotion of positive emotions depends entirely on direct effects.

**Discussion:**

These findings underscore Tai Chi’s unique capacity to both directly enhance wellbeing and empower older adults to manage negative emotions through improved emotional regulation. To translate these findings into practice, community health programs should integrate Tai Chi as a low-cost, accessible intervention for older adults, particularly those experiencing psychological stress or chronic conditions.

## 1 Introduction

With the increasing global life expectancy, the concept of health has evolved from traditional physiological dimensions to encompass the multidimensional integration of psychological and social adaptation. Within this paradigm shift, mental health challenges among older adults are escalating into a global public health crisis at an alarming rate. Studies demonstrate that depression and anxiety—the most prevalent mental health disorders in older populations—exhibit exponentially rising incidence rates with advancing age (Sharma et al., 2021), while societal structural shifts (e.g., nuclear family dynamics and population mobility) further exacerbate loneliness and social isolation among the elderly (Luo et al., 2024). This multidimensional mental health crisis forms a vicious cycle with the progressive deterioration of subjective wellbeing: empirical studies have demonstrated that older adults’ subjective wellbeing is significantly correlated with their mental health status. Specifically, those with lower levels of subjective wellbeing exhibit heightened vulnerability to depressive symptoms and are at a markedly elevated risk of suicidal behaviors (Sharma, 2022; Zhu et al., 2025). The advent of the COVID-19 pandemic in 2020 has not only jeopardized physical health but has also engendered a surge in psychological maladies, including anxiety and depression (Bodecka et al., 2021). In this context, tackling the challenges incumbent upon an aging society and ameliorating the pandemic’s ramifications on the mental health of older adults have become exigent research imperatives. This study, therefore, endeavors to uncover efficacious strategies for the amelioration of mental health and the enhancement of subjective wellbeing among older adults, aligning with the contemporary societal imperatives.

With the development of sport psychology, an increasing body of research indicates that engaging in regular physical activity has a significant positive impact on an individual’s subjective wellbeing. For instance, Kye and Park (2014) found through a random sample survey of 1,530 adults aged 30–69 that those who exercised at least five times per week for no less than 30 min each time reported higher levels of happiness. Similarly, a study focusing on 3,461 university students (aged 17–24) in Chile observed analogous results: students participating in daily physical activities were more likely to exhibit higher indices of wellbeing (Piqueras et al., 2011). Moreover, Moljord et al.’s (2011) investigation into 1,508 adolescents (aged 13–18) in Norway revealed a clear association between frequent participation in sports activities and higher levels of happiness. Wang et al. (2012), using data from the Canadian Community Health Survey, pointed out that recreational physical activities not only reduce the likelihood of unhappiness but may also gradually enhance individuals’ wellbeing over time.

Though existing evidence strongly suggests that physical activity positively influences subjective wellbeing, the majority of research in this domain is cross-sectional, precluding definitive causality determinations. There is likely a reciprocal relationship between physical activity and wellbeing: physical activity can enhance health status, and improved health can, in turn, motivate increased physical activity, establishing a virtuous cycle (van Woudenberg et al., 2020). Studies have confirmed that wellbeing influences an individual’s intention to engage in physical activity (Catellier and Yang, 2013). To elucidate the causal direction between physical activity and subjective wellbeing, intervention-based research methods are essential to track changes in subjective wellbeing post-physical activity introduction.

Compared to other forms of physical activity, Tai Chi involves slow and gentle movements. Its low-impact and low-load characteristics reduce the risk of injury, making it suitable for individuals of all ages and fitness levels, particularly older adults and those with chronic conditions (Jiao et al., 2023). Secondly, exercise intervention studies targeting older adults often encounter challenges such as poor adherence and elevated dropout rates (Brawley et al., 2003). These issues are primarily due to older adults’ reluctance to engage frequently in activities they perceive as unenjoyable or inappropriate. However, Tai Chi is not merely a form of physical activity but also an integral part of traditional Chinese culture. Rooted in philosophical principles such as the balance of Yin and Yang and the harmony between mind and body, it embodies profound cultural significance. This cultural richness has contributed to Tai Chi’s widespread popularity across the globe (Dong et al., 2025). In response to these challenges, we opted for Tai Chi as our intervention method. This study formulates the following hypothesis (H1): Tai Chi training can significantly enhance the subjective wellbeing of older adults.

Emotional regulation efficacy describes a person’s belief in their capacity to handle negative emotions. It involves three key abilities: (1) staying calm under stress, (2) stopping negative thoughts from escalating, and (3) using practical strategies—like positive self-talk or deep breathing—to recover a positive mood after emotional challenges (Muris, 2002). Studies have shown a significant correlation between physical exercise and emotional regulation efficacy, indicating that higher levels of physical activity are associated with greater emotional regulation efficacy (Wang et al., 2022; Valois et al., 2008). Given that Tai Chi is a low-intensity aerobic exercise suitable for older adults, it not only benefits physical health but may also be particularly effective in enhancing emotional regulation efficacy. Therefore, this study posits the following hypothesis (H2): Tai Chi practice can improve the emotional regulation efficacy of older adults.

Research indicates that individuals with high emotional regulation efficacy typically exhibit greater life confidence and maintain higher levels of subjective wellbeing (Extremera and Rey, 2015). Conversely, those with lower emotional regulation efficacy are more likely to doubt their abilities and may opt to give up when faced with challenges, leading to increased negative emotions and a consequent reduction in subjective wellbeing (Wiesmann and Hannich, 2014). Physical exercise not only directly enhances subjective wellbeing but also strengthens emotional regulation efficacy, which in turn significantly and positively predicts wellbeing. Therefore, this study posits the following hypothesis (H3): Tai Chi improves the subjective wellbeing of older adults by enhancing their emotional regulation efficacy.

To address these gaps, the present study pursues two interrelated aims: (1) To examine the direct effects of an 8 weeks Tai Chi intervention on emotional regulation efficacy and subjective wellbeing (including positive and negative affect) in older adults. (2) To test the mediating role of emotional regulation efficacy in the relationship between Tai Chi practice and improvements in subjective wellbeing.

## 2 Materials and methods

### 2.1 Participants

This study recruited 60 healthy elderly participants from Zhengzhou City, Henan Province, China. Participants were randomly assigned to the Tai Chi group (*n* = 30) or control group (*n* = 30) using a computer-generated simple randomization sequence. The intervention was conducted in October 2023 (autumn season). The inclusion criteria were as follows: (1) aged 60 years or older; (2) no symptoms of spinal pain; (3) no confirmed structural deformities. Participants were excluded if they met any of the following conditions: (1) significant cognitive impairment; (2) acute injuries or severe cardiovascular, pulmonary, renal, or musculoskeletal diseases; (3) presence of trauma, hemiplegia, amputation, or similar conditions; (4) experience in Tai Chi or participation in other rehabilitation programs during the study period. Given the limited sample size (*N* = 60 participants total), we prioritized the collection of core demographic variables (age, BMI) and primary outcomes (emotional regulation efficacy, subjective wellbeing). Gender distribution was not statistically analyzed due to insufficient sample power to detect potential gender-related differences. To ensure baseline equivalence, we conducted post-allocation comparisons of key demographic, which revealed no significant differences between groups (all *p* > 0.05; see Table 1).

**TABLE 1:** Baseline characteristics of participants.

Baseline characteristics	Control group (*N* = 30)	Experimental group (*N* = 30)	t	*P*
Age (years)	64.90 ± 1.73	65.17 ± 1.82	–0.582	0.563
Height (m)	1.68 ± 0.08	1.71 ± 0.08	–1.288	0.203
Weight (kg)	68.77 ± 5.29	69.76 ± 6.25	–0.663	0.51
BMI (kg/m^2^)	24.40 ± 2.77	24.00 ± 3.06	0.525	0.602

### 2.2 Procedure

This study randomly assigned 60 healthy older adults into an experimental group and a control group, with 30 participants in each. All participants were informed that the study aimed to “explore health-promoting activities for older adults” but were not disclosed specific hypotheses about Tai Chi’s psychological benefits. To minimize expectancy bias, outcome assessments were conducted by trained research assistants blinded to group allocation. Prior to the start of the experiment, baseline measurements of emotional regulation efficacy and subjective wellbeing were conducted using the Emotional Regulation Efficacy Scale and the Subjective Wellbeing Scale for both groups. This ensured that no significant differences existed between the groups at the outset. If initial measurements indicated significant differences, re-randomization was performed until equivalence was achieved.

The experimental group received one-on-one instruction from a professional Tai Chi instructor to ensure that all participants correctly mastered the basic movements of Tai Chi. Following this preparatory phase, participants underwent an 8 weeks standardized 24-form simplified Tai Chi program (derived from Yang style) between October and December 2023. Each weekly session included: (1) Structure: 5 min warm-up (joint mobilization), 20 min core practice (e.g., “Commencement Form,” “Grasp the Sparrow’s Tail”), and 5 min cool-down (mindfulness breathing). (2) Dosage: Five 30 min sessions weekly, supervised by the same instructor.

During the training period, participants were instructed to maintain their usual lifestyle habits outside of the Tai Chi sessions.

After the 8 weeks intervention, both groups were reassessed using the Emotional Regulation Efficacy Scale and the Subjective Wellbeing Scale to measure changes in emotional regulation efficacy and subjective wellbeing.

### 2.3 Materials

The Emotional Regulation Efficacy Scale was selected based on its validated application in Chinese elderly populations, comprising seven items quantified on a five-point Likert scale (1 = Not at all; 5 = Very well), with total scores ranging from 7 to 35 points. Higher scores indicate stronger emotional regulation efficacy (Yujia et al., 2022). A recent study by Yujia et al. (2022) demonstrated strong psychometric properties of this scale among 9,701 older adults in China, reporting excellent internal consistency and significant correlations with activities of daily living (ADL) scores (β = 0.097, *p* < 0.013), confirming its relevance to geriatric self-efficacy. The seven-item structure (e.g., “I can manage negative emotions effectively”) directly aligns with the emotion regulation challenges prevalent in aging populations. Reliability testing of this study indicate showed Cronbach’s α values exceeding 0.75, indicating good internal consistency. Confirmatory factor analysis results demonstrated satisfactory structural validity for the scale (T1: χ^2^/df = 1.86, CFI = 1.00, TLI = 1.04, RMSEA = 0.00; T2: χ^2^/df = 1.19, CFI = 0.97, TLI = 0.95, RMSEA = 0.06).

The Subjective Wellbeing Scale was chosen for its robust validation in Chinese elderly cohorts. In a large-scale investigation of chronic illness impacts on wellbeing (Dong et al., 2025), this scale demonstrated high reliability and discriminant validity between healthy elders and those with chronic conditions. Its dual focus on positive affect (e.g., “I feel content with my life”) and negative affect (reverse-scored items like “I often feel lonely”) provides a comprehensive assessment of wellbeing, consistent with our study’s multidimensional framework. Reliability testing of this study indicate showed Cronbach’s α values exceeding 0.75, indicating good internal consistency. Confirmatory factor analysis results demonstrated satisfactory structural validity for the scale (T1: χ^2^/df = 2.21, CFI = 0.93, TLI = 0.91, RMSEA = 0.06; T2: χ^2^/df = 2.24, CFI = 0.91, TLI = 0.95, RMSEA = 0.05).

### 2.4 Data processing and analysis

Following the longitudinal mediation effect testing procedure proposed by Jie et al. (2021) we set group assignment as the independent variable X, the difference in emotional regulation efficacy before and after intervention as the mediator M, and the differences in positive affect, negative affect, and subjective wellbeing before and after intervention as the dependent variables Y, to explore the underlying mechanisms by which Tai Chi practice improves subjective wellbeing.

Data analysis was conducted using SPSS version 25.0. Means and standard deviations were calculated for all variables. For data that conformed to a normal distribution, paired *t*-tests were used for within-group comparisons; for data not conforming to a normal distribution, non-parametric Mann-Whitney U tests were employed. Between-group comparisons were conducted using independent samples *t*-tests for normally distributed data and non-parametric Mann-Whitney U tests for non-normally distributed data. Reliability and validity of the questionnaires were assessed through reliability testing and exploratory factor analysis. Mediation effects were examined using Model 4 in the Process macro for SPSS. The significance level was set at *p* < 0.05.

## 3 Results

### 3.1 Baseline equivalence

To guarantee the validity and comparability of the experimental outcomes, an assessment of homogeneity was conducted between the two groups concerning emotional regulation efficacy and subjective wellbeing prior to the intervention. As indicated in Table 2, no significant differences were observed between the groups with respect to emotional regulation efficacy, positive affect, negative affect, and subjective wellbeing before the intervention.

**TABLE 2:** Baseline equivalence between groups.

Variable	Control group (*N* = 30)	Experimental group (*N* = 30)	t	*P*
Emotional regulation efficacy	23.37 ± 2.92	23.60 ± 2.33	–0.342	0.733
Positive affect	27.77 ± 3.31	27.53 ± 2.46	0.31	0.758
Negative affect	30.27 ± 3.34	30.70 ± 2.90	–0.536	0.594
Subjective wellbeing	58.03 ± 6.59	58.23 ± 5.29	–0.13	0.897

### 3.2 Within-group comparisons

To assess the impact of the Tai Chi intervention, within-group comparisons were performed for both the control and experimental groups, focusing on emotional regulation efficacy, positive affect, negative affect, and subjective wellbeing, both prior to and following the intervention. The findings are detailed in Table 3. The control group exhibited no significant changes in any of the measured variables. Conversely, the experimental group showed significant post-intervention improvements in emotional regulation efficacy (23.60 ± 2.33 to 26.60 ± 3.10, *p* < 0.001, Cohen’s d = 1.29), positive affect (27.53 ± 2.46 to 29.43 ± 3.12, *p* < 0.001, Cohen’s d = 0.77), negative affect (30.70 ± 2.90 to 32.13 ± 3.05, *p* = 0.002, Cohen’s d = 0.49), and subjective wellbeing (58.23 ± 5.29 to 61.57 ± 5.04, *p* = 0.000, Cohen’s d = 0.63).

**TABLE 3:** Within-group comparisons before and after intervention.

Variable	Pre-intervention	Post-intervention	t	*P*	Cohen’s d
Emotional regulation efficacy	Control: 23.37 ± 2.92	Control: 24.30 ± 2.87	–1.159	0.256	0.32
	Exp.: 23.60 ± 2.33	Exp.: 26.60 ± 3.10	–5.326	0	1.29
Positive affect	Control: 27.77 ± 3.31	Control: 27.23 ± 2.97	1.23	0.228	–0.16
	Exp.: 27.53 ± 2.46	Exp.: 29.43 ± 3.12	–3.266	0	0.77
Negative affect	Control: 30.27 ± 3.34	Control: 30.20 ± 2.56	0.239	0.813	–0.02
	Exp.: 30.70 ± 2.90	Exp.: 32.13 ± 3.05	–3.37	0.002	0.49
Subjective wellbeing	Control: 58.03 ± 6.59	Control: 57.43 ± 5.15	1.048	0.303	–0.09
	Exp.: 58.23 ± 5.29	Exp.: 61.57 ± 5.04	–5.49	0	0.63

### 3.3 Between-group comparisons

Further evaluation of the Tai Chi intervention’s effect involved comparing the two groups post-intervention regarding emotional regulation efficacy, positive affect, negative affect, and subjective wellbeing. As indicated in Table 4, compared to the control group, the experimental group showed significantly greater improvements in emotional regulation efficacy (24.30 ± 2.87 to 26.60 ± 3.10, *p* = 0.004, Cohen’s d = 0.77), positive affect (27.23 ± 2.97 to 29.43 ± 3.12, *p* = 0.007, Cohen’s d = 0.72), negative affect (30.20 ± 2.56 to 32.13 ± 3.05, *p* = 0.010, Cohen’s d = 0.69), and subjective wellbeing (57.43 ± 5.15 to 61.57 ± 5.04, *p* = 0.003, Cohen’s d = 0.81).

**TABLE 4:** Between-group comparisons post-intervention.

Variable	Control group	Experimental group	t	*P*	Cohen’s d
Emotional regulation efficacy	24.30 ± 2.87	26.60 ± 3.10	–2.982	0.004	0.77
Positive affect	27.23 ± 2.97	29.43 ± 3.12	–2.802	0.007	0.72
Negative affect	30.20 ± 2.56	32.13 ± 3.05	–2.664	0.01	0.69
Subjective wellbeing	57.43 ± 5.15	61.57 ± 5.04	–3.141	0.003	0.81

### 3.4 Mediation analysis

**FIGURE 1:**
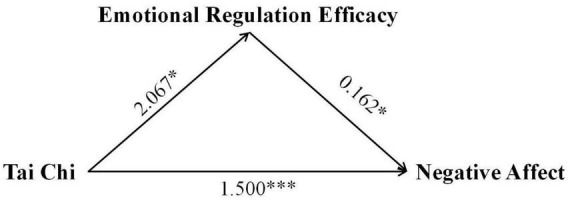
Path coefficients diagram of Tai Chi, emotional regulation efficacy, and positive affect. *p < 0.05, ***p < 0.001.

**FIGURE 2:**
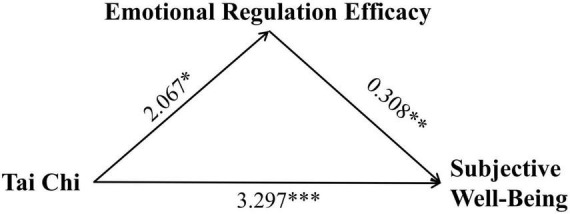
Path coefficients diagram of Tai Chi, emotional regulation efficacy, and negative affect. *p < 0.05, **p < 0.01, ***p < 0.001.

**FIGURE 3:**
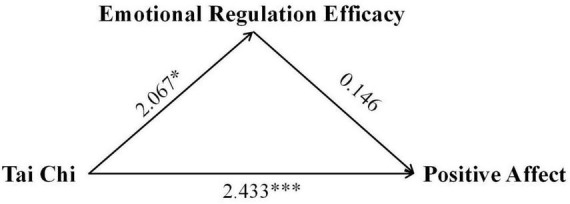
Path coefficients diagram of Tai Chi, emotional regulation efficacy, and subjective wellbeing. *p < 0.05, ***p < 0.001.

To gain a deeper understanding of the long-term impact pathways of Tai Chi practice on emotional regulation efficacy and subjective wellbeing, we constructed and tested a mediation model. The study designated Tai Chi practice intervention as the independent variable, the change in emotional regulation efficacy as the mediator, and the changes in positive affect, negative affect, and subjective wellbeing as the dependent variables. This analytical framework aimed to reveal how Tai Chi practice influences emotional states and life satisfaction by affecting emotional regulation efficacy. The mediation analysis demonstrated that Tai Chi’s benefits operated through both direct and indirect pathways, see Table 5. Crucially, emotional regulation efficacy mediated 22.27% of Tai Chi’s total effect on reducing negative affect [indirect effect: 0.334, 95% CI (0.005, 0.761)] (see Figure 1) and 16.20% of its effect on subjective wellbeing [indirect effect: 0.637, 95% CI (0.002, 1.731)] (see Figure 2). In contrast, no mediation was observed for positive affect, underscoring that Tai Chi’s enhancement of positive emotions relies solely on direct mechanisms (see Figure 3).

**TABLE 5:** Mediation analysis of Tai Chi practice on emotional regulation efficacy and subjective wellbeing.

Dependent variable	Effect type	Effect value	SE	LLCI	ULCI	Proportion of total effect	Significance
Positive affect	Total effect	2.433	0.631	1.171	3.696	–	Significant
	Direct effect	2.131	0.643	0.844	3.418	87.59%	Significant
	Indirect effect	0.303	0.308	–0.072	1.069	12.45%	Not significant
Negative affect	Total effect	1.5	0.509	0.482	2.519	–	Significant
	Direct effect	1.166	0.506	0.153	2.179	77.73%	Significant
	Indirect effect	0.334	0.193	0.005	0.761	22.27%	Significant
Subjective wellbeing	Total effect	3.933	0.834	2.263	5.604	–	Significant
	Direct effect	3.297	0.814	1.667	4.926	83.83%	Significant
	Indirect effect	0.637	0.453	0.002	1.731	16.20%	Significant

## 4 Discussion

In the realm of exercise science, the effects of exercise interventions on subjective wellbeing and emotional regulation efficacy have increasingly become central topics of investigation. The nexus between emotional regulation efficacy and subjective wellbeing has particularly piqued scholarly interest. Despite an abundance of evidence highlighting the salutary effects of physical activity on these constructs, a systematic exploration into the specific ways in which targeted exercise interventions might shape the relationship between emotional regulation efficacy and subjective wellbeing is lacking. To bridge this gap in the literature, the present study is designed to evaluate the impact of an 8 weeks Tai Chi training regimen on emotional regulation efficacy and subjective wellbeing in older adults, while also assessing the mediating role of emotional regulation efficacy in this dynamic.

### 4.1 Impact on subjective wellbeing

One of the objectives of this study was to investigate the impact of an 8 weeks Tai Chi training program on the subjective wellbeing of older adults. According to the data presented in Tables 2–4, the results indicate that the 8 weeks Tai Chi training significantly enhanced positive affect, reduced negative affect, and improved overall wellbeing in the experimental group. These findings are consistent with existing literature, which indicates that Tai Chi can significantly improve physical fitness and cognitive function in older adults, thereby enhancing their subjective wellbeing (Wang et al., 2023). A systematic review and meta-analysis has demonstrated that Tai Chi effectively improves both mental and physical health in patients with depression, alleviating symptoms of depression and anxiety while enhancing quality of life (Sani et al., 2023). Similarly, for adults with cardiovascular diseases, Tai Chi has been shown to significantly improve mental health, elevate quality of life, and reduce depression and psychological stress (Taylor-Piliae and Finley, 2020). This study further substantiates the conclusion that exercise can effectively enhance wellbeing.

Regarding the mechanisms through which Tai Chi enhances wellbeing, several hypotheses can be inferred from existing literature. The first hypothesis focuses on physiological factors. Physical activity enhances the transmission of monoamine neurotransmitters in the brain, such as norepinephrine, dopamine, and serotonin, and increases the production of endorphins. These physiological responses are known to alleviate symptoms of depression and anxiety while promoting overall wellbeing (Wasserman, 2019; Kandola et al., 2019; Phillips and Fahimi, 2018). Research indicates that moderate physical activity not only improves mood but also affects mental health by regulating neurotransmitter levels (Balchin et al., 2016; Wolff et al., 2011; Eyre and Baune, 2012). Additionally, physical activity is associated with immune function and neuroprotective effects, which play a crucial role in the pathophysiology of depression (Eyre and Baune, 2012). van Woudenberg et al.’s (2020) study on the relationship between adolescents’ physical activity and happiness demonstrated reciprocal short-term impacts: adolescents feel happier when they are more active, and they are more willing to engage in physical activity within the following hours when they feel happy. However, this reciprocal relationship is not evident over several days (van Woudenberg et al., 2020), which aligns more with physiological processes that occur within 30 min to 1 h after exercise (Farrell et al., 1982).

The second hypothesis zeroes in on psychological factors. Engaging in physical exercise enables individuals to derive greater pleasure and a sense of pride from movement, which in turn significantly bolsters their wellbeing (Gilchrist et al., 2018). Taking part in sports also offers social benefits, such as increased social interaction and engagement (Smith, 2003), thereby providing participants with positive happiness experiences (Downward and Rasciute, 2011; Becchetti et al., 2008). For example, data from the Scottish Health Survey suggest that recreational sports are more effective at enhancing wellbeing than household chores (Hamer et al., 2009). A study conducted in Belgium similarly found that happiness correlates positively with recreational sports and negatively with household chores (Asztalos et al., 2009). A comparative analysis of recreational sports versus other forms of exercise has shown that recreational sports are more effective in boosting wellbeing (White et al., 2018). Moreover, researchers have observed that excessive exercise might lead to a decrease in wellbeing, implying that the enhancement of happiness could be tied to the enjoyment individuals derive from exercising (Wilson et al., 2022). These studies underscore the substantial role of psychological factors in the mechanisms by which exercise enhances wellbeing.

Finally, cultural factors may play a critical role in the mechanism by which Tai Chi interventions enhance wellbeing. As a traditional Chinese martial art, Tai Chi is not merely a form of physical exercise but also a cultural practice deeply rooted in Eastern philosophy. The practice of Tai Chi emphasizes inner peace and mindfulness, aligning with the pursuit of mind-body harmony central to many Eastern cultures. Consequently, for individuals from these cultural backgrounds, Tai Chi may be more readily accepted and more effective in improving their wellbeing. Research indicates that Tai Chi interventions can significantly enhance self-esteem in adults—a phenomenon potentially mediated by cultural influences on self-perception (Chair et al., 2025).

### 4.2 Implications for enhancing emotional regulation efficacy

Another pivotal aim of this study was to assess the impact of an 8 weeks Tai Chi training program on the emotional regulation efficacy of older adults. As per Table 2, initial comparisons revealed no significant disparities in emotional regulation efficacy between the experimental and control groups, ensuring baseline equivalence. Post-intervention, however, the data from Tables 3, 4 indicate a marked enhancement in emotional regulation efficacy within the experimental group following the 8 weeks Tai Chi regimen. This outcome is consistent with extant literature highlighting the positive association between physical activity and emotional regulation efficacy (Wang et al., 2022; Valois et al., 2008; Downs and Strachan, 2016).

Sport psychology research has consistently shown a synergistic relationship between engagement in physical exercise and emotional regulation efficacy. Active participation in sports activities has been found to positively influence individuals’ ability to regulate their emotions, with efficacy often escalating alongside increased exercise duration and intensity. This heightened emotional regulation efficacy is linked to greater life satisfaction and has been shown to foster both mental and physical health (Wang et al., 2022; Valois et al., 2008; Downs and Strachan, 2016). Despite this, the majority of current studies adopt a cross-sectional design, which poses challenges in establishing a causal link between physical activity and emotional regulation efficacy.

By controlling for confounding variables and treating exercise participation as the sole independent variable, this study further elucidates the causal impact of exercise participation on emotional regulation efficacy. The longitudinal design of our study provides stronger evidence for the causal relationship compared to previous cross-sectional studies. Our findings suggest that engaging in Tai Chi not only improves emotional regulation efficacy but also offers insights into the mechanisms by which regular physical activity can enhance psychological resilience and wellbeing in older adults.

### 4.3 Mediating role of emotional regulation efficacy

This study reveals that emotional regulation efficacy significantly mediates the effects of Tai Chi on reducing negative affect and enhancing wellbeing, a role not observed for positive affect. The differential mediating effects of emotional regulation efficacy on positive versus negative emotions may stem from the inherent nature of emotion regulation as a self-regulatory capacity to manage adversity. Emotional regulation efficacy denotes an individual’s confidence in managing their emotions effectively. Individuals with lower emotional regulation efficacy may struggle to self-motivate and maintain efforts toward goal achievement when confronted with challenging tasks (Schmidt and Pichler, 2020). In contrast, those with higher emotional regulation efficacy are more likely to perceive difficulties as challenges and opportunities, rather than threats (Bandura, 2012). This constructive outlook not only improves performance but also mitigates stress and its detrimental impacts. This attribute may underpin the mediating role of emotional regulation efficacy in the amelioration of negative affect and wellbeing through Tai Chi practice.

Specifically, throughout the course of Tai Chi training, participants incrementally build their confidence in emotional management by mastering and applying emotional regulation techniques. This heightened emotional regulation efficacy equips them with improved strategies to navigate negative emotions in their daily lives, consequently elevating their overall wellbeing. Nevertheless, the influence on positive affect appears to be less dependent on emotional regulation efficacy alone and may encompass additional mechanisms, such as the instantaneous pleasure associated with physical activity or other psychological factors.

Tai Chi, as a gentle form of physical exercise, is particularly well-suited for the elderly population. Given its significant positive impact on subjective wellbeing, emotional regulation efficacy, and positive affect, community health programs can incorporate Tai Chi into their daily activity schedules to promote the overall wellbeing of older adults. Specifically, community centers can implement Tai Chi programs in the following ways. First, by organizing regular Tai Chi classes and inviting professional instructors to ensure the accuracy and safety of the movements. Second, by integrating educational lectures that introduce the psychological and physiological benefits of Tai Chi, thereby enhancing participants’ understanding and interest. Finally, by encouraging group practice sessions to foster social interaction, reduce feelings of loneliness, and further improve mental health.

Despite Tai Chi’s growing popularity as a mind-body intervention, critical gaps persist in the literature. First, prior research on Tai Chi’s mental health benefits often lacks longitudinal designs. Second, cultural factors influencing Tai Chi’s efficacy (e.g., familiarity with Eastern practices) remain underexplored, as 89% of trials have been conducted in East Asian populations. Our study directly addresses these gaps by: (1) Employing an 8 weeks longitudinal design to track dynamic changes in emotional regulation and wellbeing. (2) Integrating mediation analysis to unravel Tai Chi’s psychological pathways—a methodological advance absent in most studies. (3) Contextualizing findings within Chinese cultural practices, offering insights for adapting Tai Chi to diverse populations.

### 4.4 Limitations and prospects

While this study yields substantial insights into the effects of Tai Chi training on emotional regulation efficacy and subjective wellbeing among older adults, it is not without limitations that warrant acknowledgment. First and foremost, the study enrolled a modest sample of 60 participants, all from a specific geographic region. This may limit the external validity of the study results, as they may not fully represent a broader elderly population. As well the small sample size which limited to conduct a subgroup analyses by gender. Another critical consideration is the potential selection bias introduced by the recruitment strategy. Participants were recruited exclusively through local community centers, which may have limited the demographic and socioeconomic diversity of the sample. Therefore, future large-scale studies should prioritize stratified sampling designs to investigate potential variations in Tai Chi’s effects on emotional regulation and wellbeing across sex, geographic regions, age groups, and socioeconomic statuses.

Additionally, this study relied on self-reported data from participants, which may be susceptible to social desirability bias, subjective bias, or recall bias, thereby affecting measurement accuracy. To bolster the reliability of the data, future studies might integrate objective physiological markers or alternative assessment techniques, including behavioral observations and psychophysiological assessments.

Thirdly, although the 8 weeks intervention period demonstrated notable effects, the long-term impacts of Tai Chi training remain obscure. Future research should incorporate extended follow-up periods to elucidate the enduring and cumulative effects of Tai Chi on the mental health of older adults.

Fourth, although all participants (both the control and Tai Chi groups) were instructed to retain their baseline lifestyle habits during the trial, potential variations in daily routines (e.g., diet, sleep patterns, or incidental physical activity) were not objectively monitored or restricted. This lack of standardized control over lifestyle factors may introduce confounding effects on the outcomes.

Lastly, while our study highlighted the mediating role of emotional regulation efficacy, the intricate mechanisms at play behind this mediation necessitate further investigation. Other potential mediators, including social support and cognitive restructuring, may also exert significant influence. Future research should delve into additional mediators beyond emotional regulation efficacy, such as cognitive restructuring and social interaction, to construct a more holistic theoretical framework elucidating the nexus between exercise and mental health.

### 4.5 Suggestions and strategies

In light of the beneficial effects of Tai Chi on enhancing emotional regulation efficacy and subjective wellbeing among the elderly, there is a compelling case for communities and medical institutions to actively advocate for and implement Tai Chi training programs. Such initiatives should be particularly targeted toward older adults who are under greater psychological duress or are managing chronic health conditions. It is crucial to encourage the formation of social support networks among the elderly through their participation in Tai Chi training, thereby increasing mutual interaction and support and cultivating a nurturing environment characterized by mutual assistance and encouragement. The regular organization of group activities or competitions can further strengthen team cohesion and foster a sense of belonging among participants.

## Conclusion

Our study concluded that an 8 weeks Tai Chi intervention significantly enhanced both the emotional regulation efficacy and subjective wellbeing among older adults. Notably, the training not only directly improved participants’ capacity to manage their emotions but also indirectly amplified their overall sense of wellbeing through the mediating effect of enhanced emotional regulation efficacy.

The evidence from this study holds significant implications for multiple professional domains. Can integrate Tai Chi into community-based mental health promotion programs for older adults, particularly in regions with limited access to clinical psychological services. Can integrate Tai Chi into community-based mental health promotion programs for older adults, particularly in regions with limited access to clinical psychological services. Can integrate Tai Chi into community-based mental health promotion programs for older adults, particularly in regions with limited access to clinical psychological services.

## Data Availability

The original contributions presented in the study are included in the article/Supplementary material, further inquiries can be directed to the corresponding author.

## References

[B1] Asztalos M. Wijndaele K. De Bourdeaudhuij I. Philippaerts R. Matton L. Duvigneaud N. (2009). Specific associations between types of physical activity and components of mental health. *J. Sci. Med. Sport* 12 468–474. 10.1016/j.jsams.2008.06.009 18768366

[B2] Balchin R. Linde J. Blackhurst D. Rauch H. Schönbächler G. (2016). Sweating away depression? The impact of intensive exercise on depression. *J. Affect. Disord.* 200 218–221. 10.1016/j.jad.2016.04.030 27137088

[B3] Bandura A. (2012). On the functional properties of perceived self-efficacy revisited. *J. Manag.* 38 9–44. 10.1177/0149206311410606

[B4] Becchetti L. Pelloni A. Rossetti F. (2008). Relational goods. *Sociability, and Happiness. Kyklos* 61 343–363. 10.1111/j.1467-6435.2008.00405.x

[B5] Bodecka M. Nowakowska I. Zajenkowska A. Rajchert J. Kaźmierczak I. Jelonkiewicz I. (2021). Gender as a moderator between present-hedonistic time perspective and depressive symptoms or stress during COVID-19 lock-down. *Pers. Individ. Dif.* 168:110395. 10.1016/j.paid.2020.110395 33012936 PMC7521869

[B6] Brawley L. R. Rejeski W. J. King A. C. (2003). Promoting physical activity for older adults: The challenges for changing behavior. *Am. J. Prev. Med.* 25 172–183. 10.1016/s0749-3797(03)00182-x 14552942

[B7] Catellier J. A. Yang Z. J. (2013). The role of affect in the decision to exercise: Does being happy lead to a more active lifestyle? *Psychol. Sport Exerc.* 14 275–282. 10.1016/J.PSYCHSPORT.2012.11.006

[B8] Chair S. Law B. Chan A. Gao R. (2025). The effect of the Tai Chi intervention on self-esteem and self-confidence perception in adult populations: A systematic review and meta-analysis. *BMC Nurs.* 24:174. 10.1186/s12912-025-02792-9 39953472 PMC11829346

[B9] Dong Y. Pang D. Xiang J. Chao G. Kuang X. (2025). Exploring the benefits of traditional Chinese exercises (Tai Chi and Qigong) on the anxiety and depression of older adults: A systematic review and meta-analysis. *Medicine (Baltimore).* 104:e41908. 10.1097/MD.0000000000041908 40128068 PMC11936652

[B10] Downs M. Strachan L. (2016). High school sport participation: Does it have an impact on the physical activity self-efficacy of adolescent males? *Int. J. Hum. Movement Sports Sci.* 4 6–11. 10.13189/saj.2016.040102

[B11] Downward P. Rasciute S. (2011). Does sport make you happy? An analysis of the well-being derived from sports participation. *Int. Rev. Appl. Econ.* 25 331–348. 10.1080/02692171.2010.511168

[B12] Extremera N. Rey L. (2015). The moderator role of emotion regulation ability in the link between stress and well-being. *Front. Psychol.* 6:1632. 10.3389/fpsyg.2015.01632 26579017 PMC4621296

[B13] Eyre H. Baune B. T. (2012). Neuroimmunological effects of physical exercise in depression. *Brain Behav. Immun.* 26 251–266. 10.1016/j.bbi.2011.09.015 21986304

[B14] Farrell P. Gates W. Maksud M. Morgan W. (1982). Increases in plasma beta-endorphin/beta-lipotropin immunoreactivity after treadmill running in humans. *J. Appl. Physiol. Respir. Environ. Exerc. Physiol.* 52 1245–1249. 10.1152/jappl.1982.52.5.1245 7096149

[B15] Gilchrist J. Pila E. Castonguay A. Sabiston C. Mack D. (2018). Body pride and physical activity: Differential associations between fitness- and appearance-related pride in young adult Canadians. *Body Image* 27 77–85. 10.1016/j.bodyim.2018.08.010 30145446

[B16] Hamer M. Stamatakis E. Steptoe A. (2009). Dose-response relationship between physical activity and mental health: The Scottish health survey. *Br. J. Sports Med.* 43 1111–1114. 10.1136/bjsm.2008.046243 18403415

[B17] Jiao Q. Meng C. He H. Li S. Xu F. Cui W. (2023). Safety and effects of a home-based Tai Chi exercise rehabilitation program in patients with chronic heart failure: Study protocol for a randomized controlled trial. *Front. Cardiovasc. Med.* 10:1237539. 10.3389/fcvm.2023.1237539 38094121 PMC10716196

[B18] Jie F. Zhonglin W. Hawjeng C. (2021). Mediation analysis of longitudinal data. *J. Psychol. Sci.* 44 989–996. 10.16719/j.cnki.1671-6981.20210431

[B19] Kandola A. Ashdown-Franks G. Hendrikse J. Sabiston C. Stubbs B. (2019). Physical activity and depression: Towards understanding the antidepressant mechanisms of physical activity. *Neurosci. Biobehav. Rev.* 107 525–539. 10.1016/j.neubiorev.2019.09.040 31586447

[B20] Kye S. Y. Park K. (2014). Health-related determinants of happiness in Korean adults. *Int. J. Public Health* 59 731–738. 10.1007/s00038-014-0588-0 25033934

[B21] Luo J. Guo Y. Tian Z. (2024). Loneliness or sociability: The impact of social participation on the mental health of the elderly living alone. *Health Soc. Care Commun.* 2024, 1–12. 10.1155/2024/5614808

[B22] Moljord I. Moksnes U. Eriksen L. Espnes G. (2011). Stress and happiness among adolescents with varying frequency of physical activity. *Percept. Mot Skills* 113 631–646. 10.2466/02.06.10.13.PMS.113.5.631-646 22185078

[B23] Muris P. (2002). Relationships between self-efficacy and symptoms of anxiety disorders and depression in a normal adolescent sample. *Pers. Individ. Dif.* 32 337–348. 10.1016/S0191-8869(01)00027-7

[B24] Phillips C. Fahimi A. (2018). Immune and neuroprotective effects of physical activity on the brain in depression. *Front. Neurosci.* 12:498. 10.3389/fnins.2018.00498 30093853 PMC6070639

[B25] Piqueras J. A. Kuhne W. Vera-Villarroel P. van Straten A. Cuijpers P. (2011). Happiness and health behaviours in Chilean college students: A cross-sectional survey. *BMC Public Health* 11:443. 10.1186/1471-2458-11-443 21649907 PMC3125376

[B26] Sani N. Yusoff S. Norhayati M. Zainudin A. (2023). Tai Chi exercise for mental and physical well-being in patients with depressive symptoms: A systematic review and meta-analysis. *Int. J. Environ. Res. Public Health* 20:2828. 10.3390/ijerph20042828 36833525 PMC9957102

[B27] Schmidt G. Pichler S. (2020). General self-efficacy and body weight: The role of race and gender. *Psychol. Rep.* 124 2476–2500. 10.1177/0033294120961072 32998657

[B28] Sharma A. (2022). Relationship between depression, social isolation, and well-being among older adults. *J. Student Res.* 11:3853. 10.47611/jsrhs.v11i4.3853

[B29] Sharma M. Bhattarai T. Sharma P. (2021). Anxiety and depression among senior citizens. *J. Nepal. Health Res. Counc.* 19 305–310. 10.33314/jnhrc.v19i2.3120 34601521

[B30] Smith A. L. (2003). Peer relationships in physical activity contexts: A road less traveled in youth sport and exercise psychology research. *Psychol. Sport Exerc.* 4 25–39. 10.1016/S1469-0292(02)00015-8

[B31] Taylor-Piliae R. E. Finley B. A. (2020). Tai Chi exercise for psychological well-being among adults with cardiovascular disease: A systematic review and meta-analysis. *Eur. J. Cardiovasc. Nurs.* 19 580–591. 10.1177/1474515120926068 32515204

[B32] Valois R. Umstattd M. Zullig K. Paxton R. (2008). Physical activity behaviors and emotional self-efficacy: Is there a relationship for adolescents? *J. Sch. Health* 78 321–327. 10.1111/j.1746-1561.2008.00309.x 18489465

[B33] van Woudenberg T. J. Bevelander K. E. Burk W. J. Buijzen M. (2020). The reciprocal effects of physical activity and happiness in adolescents. *Int. J. Behav. Nutr. Phys. Act* 17:147. 10.1186/s12966-020-01058-8 33213465 PMC7678192

[B34] Wang F. Orpana H. Morrison H. de Groh M. Dai S. Luo W. (2012). Long-term association between leisure-time physical activity and changes in happiness: Analysis of the Prospective National Population Health Survey. *Am. J. Epidemiol.* 176 1095–1100. 10.1093/aje/kws199 23171884

[B35] Wang H. Liu Y. Pei Z. Liang J. Ding X. (2023). The influence of Tai Chi exercise on the subjective well-being in the aged: The mediating role of physical fitness and cognitive function. *BMC Geriatr.* 23:636. 10.1186/s12877-023-04366-3 37814237 PMC10563265

[B36] Wang K. Li Y. Zhang T. Luo J. (2022). The relationship among college students’ physical exercise, self-efficacy, emotional intelligence, and subjective well-being. *Int. J. Environ. Res. Public Health* 19:11596. 10.3390/ijerph191811596 36141869 PMC9517190

[B37] Wasserman D. (2019). Physical activity improves mental health. *Acta Paediatr.* 108 984–985. 10.1111/apa.14772 30907022

[B38] White R. Parker P. Lubans D. MacMillan F. Olson R. Astell-Burt T. (2018). Domain-specific physical activity and affective wellbeing among adolescents: An observational study of the moderating roles of autonomous and controlled motivation. *Int. J. Behav. Nutr. Phys. Act* 15:87. 10.1186/s12966-018-0722-0 30200980 PMC6131748

[B39] Wiesmann U. Hannich H. J. A. (2014). Salutogenic analysis of the well-being paradox in older age. *J. Happiness Stud.* 15 339–355. 10.1007/s10902-013-9425-z

[B40] Wilson O. Whatman C. Walters S. Keung S. Enari D. Rogers A. (2022). The value of sport: Wellbeing benefits of sport participation during adolescence. *Int J Environ Res Public Health* 19:8579. 10.3390/ijerph19148579 35886430 PMC9324252

[B41] Wolff E. Gaudlitz K. von Lindenberger B. Plag J. Heinz A. Ströhle A. (2011). Exercise and physical activity in mental disorders. *Eur. Arch. Psychiatry Clin. Neurosci.* 261 S186–S191. 10.1007/s00406-011-0254-y 21935629

[B42] Yujia X. Xiaohua W. Yuebin X. Yuanyuan F. (2022). Association between activity of daily living and self-efficacy among the elderly. *Med. Soc.* 35 95–99. 10.21203/rs.3.rs-1600110/v1

[B43] Zhu S. Liao Q. Yuan H. (2025). Effect of strength-based narrative therapy on depression symptoms and quality of life in the elderly. *Iran. J. Public Health* 54 124–132. 10.18502/ijph.v54i1.17582 39902370 PMC11787837

